# Self-serving beliefs about science: Science justifies my weaknesses (but not other people’s)

**DOI:** 10.1177/09636625241261320

**Published:** 2024-07-30

**Authors:** Francisco Cruz, André Mata

**Affiliations:** Universidade de Lisboa, Portugal

**Keywords:** folk epistemology, motivated reasoning, motivated rejection of science, rationalization, self-other differences, self-serving bias

## Abstract

This research explored the strategic beliefs that people have about science and the extent to which it can explain moral and immoral behaviors. Although people do not believe that science is able to explain certain aspects of their mind, they might nevertheless accept a scientific explanation for their immoral behaviors if that explanation is exculpatory. In a first study, participants reflected on moral and immoral deeds that they performed or that other people performed. Participants were somewhat skeptic that science can account for people’s behavior—*except* for when they reflected on the wrongdoings that they committed. Two further studies suggest that strategic belief in science arises because it enables external attributions for the behavior, outside of the wrongdoers’ control. Implications are discussed for science understanding and communication.

## Lay beliefs about science

People have lay scientific theories about how the world works. They are lay physicists (Kubricht et al., 2017), economists (Boyer and Petersen, 2018), biologists (Solomon and Zaitchik, 2012), and so on. In addition, people also have lay theories about how the mind works (e.g. Balcetis and Dunning, 2013; Chiu et al., 1997; Heath, 1999; Ong et al., 2015; Tamir et al., 2007), and what psychological phenomena science can and cannot explain (Gottlieb and Lombrozo, 2018; Mata et al., 2020). In this article, we explore whether people think that science can explain morality, in particular, whether they believe that science can explain why people (themselves included) perform certain moral and immoral behaviors, and we investigate the functions and consequences of those beliefs.

Recent research has shown that there are limits to what people believe science can explain about the mind (Gottlieb and Lombrozo, 2018), particularly their own mind (Mata et al., 2020). Thus, one might expect that people reject the idea that there is a scientific explanation for their (im)moral deeds. However, a great deal of research on motivated reasoning shows that people are strategic in how they think about science, and that they are flexible in how they approach scientific claims and evidence depending on whether they serve their preferred conclusions or not (e.g. Greitemeyer, 2014; Kahan et al., 2017; Mata et al., 2015; Munro and Ditto, 1997; Munro and Munro, 2014; Scurich and Shniderman, 2014). We predict that people might also be strategic in whether they reject or accept the notion that science can explain moral behavior. In particular, people might be more open to the possibility of science explaining their immoral behavior than their moral behavior, as a scientific explanation could offer a justification for their misdeed and thus excuse it in a sense.

## Self-serving scientific explanations

There is a long tradition of research on defensive attribution and the self-serving bias, whereby people seek to explain away their failures and misdeeds by attributing them to external causes that locate the responsibility for those shortcomings in factors that do not reflect the person’s morality and ability (Campbell and Sedikides, 1999; Riess et al., 1981; Snyder et al., 1976; for a meta-analysis, see Mezulis et al., 2004). This is one of several psychological resources that people have to construct and protect a positive self-image (Alicke and Sedikides, 2011).

A scientific explanation might work as a justification, a way for the wrongdoer to attribute the causes of their deed externally, and thus alleviate their guilt. Successes and good deeds, however, tend to be attributed internally to one’s skills and character (e.g. Steimer et al., 2019). People want the perceived responsibility for such deeds to fall on them. To the extent that a scientific explanation is seen as removing some of the responsibility for the deed (Miller et al., 1999; Newman and Bakina, 2009), people might be more averse to accepting it, as it would diminish their credit for that deed.

Indeed, there is evidence suggesting that scientific explanations can diminish perceived responsibility and control. Several studies show that the stigma and perceived blame for certain diseases and clinical conditions diminish upon learning about scientific explanations (e.g. genetic, neurobiological) for those conditions (e.g. Lebowitz and Appelbaum, 2017; Phelan et al., 2002), and the reduction of perceived blame occurs precisely because such explanations are associated with the perception that the person whose condition is explained has less control over it (Lebowitz and Appelbaum, 2017). Moreover, informing people about the neurobiological foundations of human behavior reduces their endorsement of retributive punishment of criminals (Shariff et al., 2014). And indeed, crimes justified through biogenetic conditions have been shown to receive reduced sentencing (Aspinwall et al., 2012).

## Overview

The research reviewed above shows that a reduced belief in free will (prompted by mechanistic scientific explanations) reduces one’s attribution of blame and desire to punish another person. In the present studies, participants are the agents of immoral deeds, not the victims or observers of those deeds, which should predispose them to a motivated acceptance of science (Study 1), to the extent that scientific explanations are perceived as mitigating their responsibility for the wrongdoing (Study 2).

Moreover, if this motivated reasoning account of people’s beliefs about science holds, while people might be motivated to accept the possibility of scientific explanations for their bad deeds (though not for their good deeds), when personal relevance is removed from the equation, those motivated beliefs might abate. Thus, people should be open to the thought of a scientific explanation for their bad deeds, but not for the bad deeds of other people, because in that case, there is no need to justify the deed nor any guilt to alleviate. Interestingly, research shows that, in general, and morality aside, people are more skeptical that science can explain their mind than that it can explain the mind of other people (Mata et al., 2020). However, we suspect that the opposite might hold when people reflect on whether science can account for immoral actions; in this case, they might be more open to the possibility of science explaining their behavior than that of others.

The plan of the studies is as follows: in Study 1, participants thought of good deeds or bad deeds performed by themselves or other people, and they were asked about the extent to which science can explain those deeds. We predict that people will be more open to the idea that science can explain bad deeds than good deeds, but this difference should only hold for their deeds, not those of other people. Study 2 explored the psychological consequences of scientific explanation. Participants were asked to imagine that science either could or could not explain their bad deeds, and then we assessed their understanding of those deeds. We predict that, when a scientific explanation is said to exist for people’s bad deeds, they will make more external attributions about those deeds (i.e. to situational circumstances, as opposed to attributing it to internal dispositions), they will perceive less control over their actions, and feel less guilty about them.

## Implications for science communication and public understanding of science

Our work explores people’s reactions to scientific explanations about good and bad deeds in general, in the hope of laying out a general principle about when and why people might accept versus reject science. However, we believe that this principle applies to how people react to science in a host of specific and consequential domains.

Indeed, a great deal of research has been conducted on the motivated rejection of science by the lay audience (for reviews, see Hornsey, 2020; Lewandowsky and Oberauer, 2016). For instance, certain segments of the population, in particular, are skeptical about climate change and they oppose policies and measures aimed at reducing that problem. However, if the messages alerting to the problem and suggesting solutions are framed in a way that does not clash with their interests and values, they are much more effective in changing attitudes and behavioral intentions (e.g. Campbell and Kay, 2014; Hornsey and Fielding, 2017).

Whereas those studies pertain to how people react to explanations provided by others, there is also research on self-generated explanations. Specifically, on how people strive to maintain a positive self-concept when their behavior goes against science-recommended practices, by rationalizing climate inaction, excessive consumption of water and energy, environmentally harmful dietary and travel habits, lack of exercise, and so on (Burger et al., 2022; Dreijerink et al., 2021; Geng et al., 2016; Gholamzadehmir et al., 2019; Ginn and Lickel, 2020; Hope et al., 2018; Iso-Ahola, 2013; Noblet and McCoy, 2018; Tiefenbeck et al., 2013; Wullenkord and Reese, 2021).

Underlying many of these cases is the same principle: people have a fundamental need to believe that they are good. Decades of psychological research have shown that some of the most basic and universal human motivations are self-protection and self-enhancement (Sedikides et al., 2015). The hypothesis guiding the present research follows the same principle and explores how it may be relevant for the public’s receptiveness to scientific explanations. Provided that people have a fundamental motivation to strive for a positive self-view, it is important that science communication efforts factor in the way that they may conflict with people’s self-appraisals—as people will be more predisposed to considering and accepting scientific explanations if they do not contradict their positive self-view. In this sense, the present research hopes to advance knowledge on the public understanding of science by considering the ways in which people strategically engage with science in accordance with their motivations. Whereas previous research has explored how the negative traits of particular individuals can stand in the way of public understanding and acceptance of science (e.g. conspiratorial mindsets, susceptibility to fake news, partisan bias; for example, Hamilton et al., 2015; Newman et al., 2022; Treen et al., 2020), this research tests whether this fundamental motivation to strive for a positive self-view can be harnessed for effective science communication.

Finally, these studies add to the growing body of research on how people’s understanding of science can differ depending on whether scientific recommendations are targeted at the self or other people (Mata et al., 2020). A different belief in science for self versus others might generate asymmetries in the choices people make for themselves and the advice they provide to others.

## 1. Study 1

Study 1 aimed to provide a first demonstration of motivated beliefs about the explanatory power of science. Thus, we manipulated the target whose behavior is described (self vs other) and its valence (moral vs immoral). We hypothesize that there will be differences in the extent to which participants believe that science can explain their deeds as a function of valence (such that their own bad deeds should be considered more explainable), but that no such differences will emerge when the behavior described is that of a distant other (i.e. acquaintance). For this purpose, we created a set of items based on previous research on people’s beliefs about the explanatory power of psychological science (see Gottlieb and Lombrozo, 2018; Mata et al., 2020).

Moreover, this study also explored whether laypeople are strategic mind–body dualists, associating bad deeds with the brain and good deeds with a non-material basis (their mind, soul, or spirit). Studies show that when behaviors are seen as having neurobiological bases, their agents are seen as less responsible for them (Miresco and Kirmayer, 2006; Shariff et al., 2014). The question that we explored with this secondary aim of the study was whether people have an intuition for this, and therefore make the reverse inference: as they wish to be seen as less responsible for their bad deeds, they might attribute those deeds to their brain, which is likely to be seen as operating according to deterministic laws outside of their control. As Gazzaniga (2005) put it, people might intuit that “People are free and therefore responsible for their actions; brains are not responsible,” and think that “Harry didn’t do it. His brain did it. Harry is not responsible for his actions.” On the contrary, people should want to take credit for their good deeds, and therefore, be less likely to attribute them to the brain and instead associate them with their soul/mind.

### Method

For all studies, all measures, manipulations, and exclusions are disclosed, as well as the method of determining the final sample size, and the data were not analyzed before collection was completed.

#### Participants

In total, 137 Portuguese psychology undergraduates participated in exchange for course credit. This exact sample size was not determined a priori. We merely sought to have at least 50 participants per cell of the design (see Simmons et al., 2018), in both this study and the next ones. The sample size for this study corresponds to the available number of students taking part in a study wave for course credit. In the following studies, we recruited exactly 50 participants per condition. Seven duplicate cases and six incomplete participations were excluded from the analysis, for a remaining total of 124 participants (*M_age_* = 20.06 years, *SD_age_* = 4.67 years; 12.9% male, 85.5% female, 1.6% other/rather not disclose).

#### Procedure

This study followed a 2 (Target: Self vs Other) × 2 (Valence of Behavior: Good vs Bad) mixed design, with the second factor manipulated within-subjects. First, participants were asked to describe a behavior (good or bad, counterbalanced between-subjects) that either they or an acquaintance of theirs had performed recently. Participants were then asked to keep in mind that behavior when rating their agreement with three statements concerning the extent to which science was capable of explaining such behavior (i.e. “Science can explain the action I described above. That is, there is a scientific explanation for this action”; “Scientists (e.g. neuroscientists, psychologists, etc.) are already capable of understanding the reason behind the behavior described above” and “The action that I described above is easily explained by science”; the response scale for all items ranged from 1—*Completely Disagree* to 9—*Completely Agree*). Participants finally indicated the extent to which they considered the behavior to be associated to a material or to an immaterial part of the agent (i.e. “Is the action you described associated with a non-material (e.g. mind, spirit, soul) or material (e.g. brain) part of [you/your acquaintance]?” 1—*Non-material* to 7—*Material*). For the original (Portuguese) wording of the items, see Supplemental Materials (Table A1). Then, participants wrote about a second behavior performed by the same agent (themselves or another person), but of opposite valence to the behavior that they described earlier, and they again completed the same measures.

### Results

The items pertaining to the behaviors’ scientific explainability had good-to-excellent reliability, depending on the condition (moral behaviors by the self: α = .92; immoral behaviors by the self: α = .84; moral behaviors by others: α = .92; immoral behaviors by others: α = .91). A repeated-measures ANOVA assessed the extent to which participants believed that science can explain the behaviors that they described (i.e. average of the three items described above), with Target (self vs other) and Valence (bad vs good behavior) as between- and within-subjects factors, respectively (see also Supplemental Materials, Table A2). There was a significant main effect of Valence, such that participants considered bad behaviors (*M* = 6.80, *SD* = 1.79) to be overall more explainable by science than good ones (*M* = 6.33, *SD* = 2.11), *F*(1, 122) = 10.99, *p* = .001, η_p_^2^ = .08. The main effect of Target was not significant, *F* < 1. More importantly, there was a significant Valence × Target interaction, *F*(1, 122) = 4.42, *p* = .037, η_p_^2^ = .04, whereas other people’s behaviors were considered to be equally explainable by science regardless of whether they were moral (*M* = 6.35, *SD* = 2.20) or immoral (*M* = 6.53, *SD* = 1.98), *t* < 1, participants’ own immoral behaviors were thought to be more explainable than their moral behaviors (*M* = 7.08, *SD* = 1.53 vs *M* = 6.31, *SD* = 2.02), *t*(59) = 3.40, *p* = .001, *d* = 1.76.

Importantly, these effects are not attributable to a bias in reporting of own behaviors (vs those of others) associated with self-presentational concerns, which would lead participants in the self condition to report behaviors that are more moral (and less immoral) than those reporting others’ (im)moral behaviors. To exclude this alternative interpretation of the data, two raters blind to the experimental hypothesis rated all reported behaviors in their moral valence through a seven-point Likert-type-like scale (from *–*3 = *Completely immoral* to *+*3 *=* *Completely moral*; 0 *=* *Amoral*), after identifiable cues regarding the target were removed. The ratings were later averaged to create a moral valence score; interrater reliability was moderate (weighed κ = .66). Although moral behaviors were more moral (*M* = 1.47, *SD* = 1.06) than their immoral counterparts (*M* = −1.42, *SD* = 0.99; *t*(123) = 18.74, *p* < .001, *d* = 2.82) as intended, no differences in moral valence emerged as a function of Target (*M_self_* = −0.07, *SD* = 2.07 vs *M_others_* = 0.13, *SD* = 1.38; *t* < 1).

A second repeated-measures ANOVA compared the extent to which the action was associated with material versus immaterial sources depending on Target and Valence (see Table A3 in the Supplemental Materials). Overall, participants rated immoral actions (*M* = 4.44, *SD* = 1.98) as more material-based than moral actions (*M* = 3.82, *SD* = 1.92), *F*(1, 122) = 11.44, *p* = .001, η_p_^2^ = .09. Neither the main effect of Target nor the Valence × Target interaction were significant; *F*(1, 122) = 1.83, *p* = .179 and *F* < 1, respectively.

The correlation between both dependent measures (i.e. whether the behavior is explainable by science and whether it is associated with a material vs immaterial source) was significant for both moral and immoral behaviors, *r*s ⩾ .34, *p*s ⩽ .001.

### Discussion

The results concerning the main hypothesis show that participants considered their immoral deeds as more explainable by science than their moral deeds, but they did not make the same differentiation for the moral versus immoral deeds of other people. This is a first piece of evidence that these might be strategic beliefs, such that participants wish their immoral deeds to be justifiable, whereas for other people this motivation is absent. Study 2 will further explore this motivated reasoning account.

An additional measure assessed the extent to which participants associated moral and immoral deeds with the brain versus an immaterial source (i.e. the soul). The results only partly supported the hypotheses. As expected, immoral deeds were associated with the brain to a larger extent than moral deeds. However, this did not differ for self versus others. The interaction that emerged for beliefs about the ability of science to explain the behavior was not observed for material-versus-immaterial ratings. Still, both measures correlated, such that greater beliefs in scientific explanation were associated with beliefs in a material (i.e. brain) foundation for the behavior.

The next studies will focus only on the key variable (belief in scientific explanation) and not on people’s lay beliefs about the material versus immaterial bases of behavior. Therefore, we briefly discuss these results here. Future research should try to further explore people’s tendency to associate good deeds to the soul/spirit and bad deeds to the brain. Interestingly, this effect was not moderated by whether the agent was the self or another person. This is preliminary evidence, and for the sake of full disclosure, our hypothesis was that there would be a moderation by target. Yet, this suggests that this association of the brain to bad deeds is not based on a motivated reasoning account whereby people wish to attribute their bad deeds to causes that do not reflect their real selves (presumably better represented by the soul/spirit) and that lay outside of their control (something that may be more associated with the brain). Indeed, research shows that when behaviors are perceived as resulting from neurobiological causes, their authors are considered to be less responsible and blameworthy (Miresco and Kirmayer, 2006; Shariff et al., 2014). Thus, the mechanism might be the same as the one that we explored in the following studies: bad deeds are seen as resulting from lack of control.

## 2. Study 2a

Study 1 provided a first demonstration of motivated beliefs about the explanatory power of science, such that individuals believe that science is more capable of explaining their bad deeds compared to their good deeds (or other people’s deeds—good or bad). Study 2’s goal is to explore why this might be the case. In this study, we explored people’s thoughts about their bad deeds (the condition that stood out in Study 1). Participants were asked to imagine that science could (or could not, in another condition) explain a bad deed of theirs, and they were asked, if that were the case, how they would feel about what they did. Specifically, participants completed items concerning external attribution, perceived control, and feelings of guilt. Our prediction is that, if science can explain their bad deeds, people will make more external attributions for those deeds, perceive less control over them, and thus feel less guilty about them. The rationale is that, to the extent that believing that one’s behavior is immoral threatens one’s self-image that should trigger negative feelings, such as guilt. If scientific explanations can deflect this threat to people’s self-concept and esteem (namely by reducing internal attributions about, and feelings of control over, one’s behavior), this should assuage such feelings of guilt.

### Method

#### Participants

One-hundred participants were recruited through Prolific, a platform for online participant recruitment in exchange for monetary compensation. Ninety-nine successfully completed the study, and ten participants were further excluded, as they failed to describe an immoral behavior or could not recall any. Therefore, our sample consisted of 89 participants (*M_age_* = 33.70 years, *SD_age_* = 10.31 years; 33.7% male, 66.3% female); participants were either American or British and had English as their first language.

#### Procedure

Participants were asked to recall and describe (by writing) an immoral behavior that they had performed recently. Then, participants were asked to imagine that science was either capable or incapable of explaining the behavior in question (i.e. “Imagine that scientists tried to explain the reasons (e.g. the underlying causes) for your behavior [and/but] they [were/were not] able to come up with a reasonable explanation for why and how you did what you did”; manipulated between-subjects), and reminded to have this scenario in mind (i.e. that there was, or was not, a scientific explanation) when answering the dependent measures (i.e. all items were preceded by “If there is [a/no] scientific explanation for what you did, that means . . .”). Eleven items were adapted from the revised Gudjonsson blame attribution inventory (Gudjonsson and Singh, 1989). We adapted the items so that they could apply to various sorts of bad deeds, and not only to severe crimes, as in the original scale (see Supplemental Materials, Table A4). All items were presented in random order and participants answered using a nine-point Likert-type-like scale. Four items measured guilt toward the behavior conducted, four items assessed attributions for the behavior and three items evaluated the perceptions of control over the behavior. Higher scores for guilt and control represent higher values of experienced guilt about, and control over, the behavior; high scores for attribution, however, represent an internal locus of responsibility (i.e. less external attribution). This applies to the coding of the variables in both Studies 2a and 2b.

### Results

Cronbach’s alphas ranged between acceptable and good (guilt: α = .90, attribution: α = .78, control: α = .83). Participants indicated that they would feel less guilt, *t*(87) = 4.01, *p* < .001, *d* = 1.40, and make more external attributions about their behavior, *t*(87) = 5.56, *p* < .001, *d* = 1.32, if there was a scientific explanation for their behavior than if there was none (*M* = 4.24, *SD* = 1.24 vs *M* = 5.45, *SD* = 1.59 and *M* = 4.34, *SD* = 1.16 vs *M* = 5.91, *SD* = 1.51, respectively; lower attribution scores indicate less internal attribution, that is, greater external attribution). The trend for perceived control was as expected, with participants indicating less perceived control over their behavior upon imagining that there was a scientific explanation for it (*M* = 5.99, *SD* = 1.74; against *M* = 6.57, *SD* = 1.90, for those who imagined there was no scientific explanation), but this difference was not significant, *t*(87) = 1.49, *p* *=* .140.

Correlations between the dependent measures show that there is a strong positive association between guilt and attribution, *r* = .84, *p* < .001, as well as a positive moderate-to-strong association between guilt and perceived control, *r* = .46, *p* < .001, and between attribution and perceived control, *r* = .44, *p* < .001.

**Figure 1.**
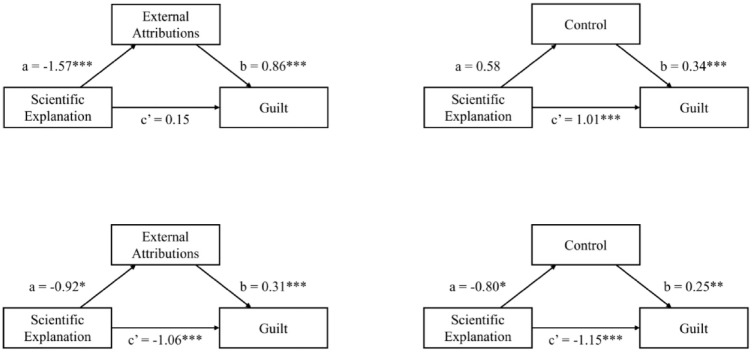
Mediation analyses of the effect of existence of a scientific explanation on guilt through external attributions (left) and control (right), for Studies 2a (top) and 2b (bottom).

Mediational analyses (using bootstrapping) explored the relation between the existence of a scientific explanation and the dependent variables. We conducted analyses for the mediation of the effect of the existence of a scientific explanation on guilt through external attributions and control separately. As predicted, external attribution mediated the effect of the manipulation (i.e. imagining that there was vs was not a scientific explanation) on guilt (indirect effect: *B* = −1.35, *SE* = 0.24, 95% CI = [–1.81, –0.86]; see Figure 1). Thus, the existence of a scientific explanation led to increased external attributions, which, in turn, decreased guilt. Consistent with the *t*-test reported above, perceived control did not mediate the relation between the manipulation and guilt (indirect effect: *B* = −0.20, *SE* = 0.14, 95% CI = [–0.51, 0.07]).

## 3. Study 2b

Study 2b tests the same hypothesis as Study 2a, while addressing some of its limitations. First, in Study 2a, the items that comprised the dependent variables (perceived control, attribution, and guilt) were selected from the same instrument (the revised Gudjonsson blame attribution inventory; Gudjonsson and Singh, 1989), which might account for the large correlations that were observed between those variables. In Study 2b, guilt, perceived control and external attribution were assessed with more items, and items belonging to different instruments, in order to test the robustness of the relations observed in Study 2a.

Second, in Study 2a, the effect of believing that there is (vs is not) a scientific explanation on perceived control was not significant. However, that dependent variable (perceived control) comprised fewer items than the other ones. Thus, in Study 2b, a larger number of items were used to measure perceived control.

### Method

#### Participants

One-hundred participants were recruited through the Prolific platform in exchange for monetary compensation. Participants were from either the United States or the United Kingdom; all spoke English as their first language. Nine participants were excluded from the analysis, as they indicated not being able to recall an immoral behavior; thus, final sample was of 91 participants (*M_age_* = 36.08 years, *SD_age_* = 11.82 years; 39.6% male, 60.4% female).

#### Procedure

Study 2b followed a procedure akin to that of Study 2a, except for two changes. First, the items pertaining to each dependent measure (guilt, attribution, and perceived control) appeared in separate blocks, with the items within each block presented in random order, and the different blocks also presented in random order. Second, the items for each block/variable came from different instruments. Guilt was assessed through the four items used in Study 2a and two additional items adapted from the same scale (Gudjonsson and Singh, 1989). External attribution was measured through two items adapted from Tamborini et al. (2018), three items adapted from Russell (1982), and one item adapted from Cucchi and Cavazza (2021). Perceived control was measured through three items from the Causal Dimension Scale (Russell, 1982), three items from the Revised Causal Dimension Scale (McAuley et al., 1992), and four items adapted from the Free Will Inventory (Nadelhoffer et al., 2014). Items were answered using a nine-point Likert-type-like scale and can be consulted in Supplemental Materials (Table A5).

### Results

Reliability was high for all scales (guilt: α = .90, external attribution: α = .85, control: α = .95). When asked to imagine that there was a scientific explanation for their actions, participants reported experiencing less guilt (*M* = 3.70, *SD* = 1.33 vs *M* = 5.04, *SD* = 1.55; *t*(89) = 4.42, *p* < .001, *d* = 1.41), perceiving less control over their actions (*M* = 6.05, *SD* = 1.92 vs *M* = 6.85, *SD* = 1.73; *t*(89) = 2.00, *p* = .048, *d* = 1.85), and they made more external attributions (*M* = 5.45, *SD* = 1.80 vs *M* = 6.37, *SD* = 1.57; *t*(89) = 2.48, *p* = .015, *d* = 1.72; once again, lower attribution scores indicate lower internal attribution and higher external attribution), relative to those who imagined that there was no scientific explanation for their actions.

Positive moderate-sized correlations were observed between guilt and attribution, *r* = .44, *p* *<* .001, such that external attribution was associated with lower guilt, and between guilt and perceived control, *r* = .37, *p* *<* .001. Attribution was strongly associated with perceived control, *r* = .77, *p* *<* .001, such that external attributions were associated with less control.

As in Study 2a, we ran mediational analyses to explore the relationship between the existence of a scientific explanation and guilt, considering external attributions and control separately. External attributions mediate the effect of the existence of a scientific explanation on guilt, such that scientific explanations inspire external attributions, which in turn reduce guilt (indirect effect: *B* = −0.28, *SE* = 0.13, 95% CI = [–0.57, –0.05]). Similarly, the effect of the existence of scientific explanations on guilt is mediated by perceived control (*B* = −0.20, *SE* = 0.11, 95% CI = [–0.45, –0.002]). Having a scientific explanation for one’s behavior is associated with a lower perception of control over that behavior, which reduces guilt.

### Discussion

Study 2 tested the effects of believing that there is a scientific explanation for one’s misdeeds on perceived control, attributions, and feelings of guilt. Results show that scientific explanations decrease perceived control (though this result was significant only in Study 2b, not in Study 2a, which probably results from Study 2a having fewer and poorer items), and they inspire external attributions, and as a result of these effects, they dampen feelings of guilt.

## 4. General discussion

These studies suggest that people have strategic beliefs about science and what it can and cannot explain. Specifically, they are more open to the notion that science can explain their immoral deeds than their moral deeds. This double standard was only observed for the self, not when thinking about the moral and immoral deeds of others, for which no ego protection is required (Study 1). And it seems to serve a defensive attributional style, whereby scientific explanations are perceived as displacing the responsibility and control for the immoral deed away from its agent (Study 2).

Recently, there have been many documented cases of motivated rejection of science (Hornsey, 2020; Lewandowsky and Oberauer, 2016). Our research documents that the opposite can occur as well: a strategic openness to scientific explanations. Just as with other cases where motivated rejection of science can be overcome when information is presented in a way that serves the audience’s desired beliefs and ideals (Bain et al., 2012; Campbell and Kay, 2014; Fielding et al., 2020; Hornsey and Fielding, 2017; Wolsko, 2017), people seem to be flexible and strategic in how they perceive science, not only rejecting it when it goes against their cherished beliefs, but also accepting it when helps to protect a positive self-image. Thus, whereas much has been discussed about the motivated rejection of science, this work sought to demonstrate that this phenomenon is broader and more nuanced than mere motivated disbelief, and that it also extends to motivated belief in, and acceptance of, science.

There is research outside the domain of lay beliefs about science, and science communication, showing that people endorse different explanations depending on what is favorable to them. For instance, in studies concerning lay attributions about the Holocaust, whereas Israeli participants are more likely to make attributions based on the evil character of Germans; German participants tend to give more credence to explanations emphasizing factors beyond the Germans’ character such as obedience and situational constraints (Doosje and Branscombe, 2003; Hirschberger et al., 2022; Imhoff et al., 2017)—which are well-known to psychological science. However, when thinking of positive actions (and not wrongdoings) of their group, the pattern reverses: people tend to make self-serving attributions focusing on internal dispositional factors rather than situational factors (Bilewicz et al., 2017). Our research shows that what holds for lay attributions about history also holds people’s lay beliefs about science: people should welcome scientific explanations that exculpate them from their wrongdoings, or that give them credit for their good deeds.

This research has implications for how to optimize communication of science to the general public. By being aware that self-regard influences the way people engage with scientific explanations, science communicators can better anticipate which topics may be met with skepticism and devise the exchange accordingly. Namely, if possible, scientific communication should avoid making its receptors feel like moral wrongdoers. For example, there is plenty of evidence now that, while people tend to believe in climate change, they do not always believe that humans are responsible for it (e.g. Campbell and Kay, 2014; Weisberg et al., 2021). Underlying this gap might be a motivated reasoning mechanism. If so, messages aimed at reducing inaction concerning climate change might be more efficient if they focus on the problem and what can be done about it, but not so much on people’s responsibility for it (for evidence consistent with this hypothesis, see Jang, 2013). Conversely, if scientific explanations for climate change inaction (of which there are several; for a review, see Hornsey and Fielding, 2020) are tailored so as to exculpate people from their previous inaction, they might be better accepted and promote action.

Moreover, while previous research trying to combat motivated rejection of undesired evidence (e.g. evidence that goes against one’s values and beliefs) finds that some techniques are able to reduce motivated rejection, sometimes that is the most they can do: reduce rejection (e.g. Bain et al., 2012; Feinberg and Willer, 2015). However, we believe that the present findings suggest the possibility that other interventions might be used to promote acceptance of evidence (as in our studies, where participants wanted to believe the evidence), and not simply reduce rejection.

It is also worthwhile to reflect on the various ways in which the mental processes underlying this motivated acceptance of scientific explanations might differ from those identified in previous research about how the public reacts to science. First, this motivated acceptance of scientific evidence does not seem to be the result of the same mind-set that is behind other lay beliefs about science—for instance, the conspiracy-prone mind-set that is behind the motivated rejection of established science and the motivated acceptance of pseudo-science, which comes from a fundamental disposition to mistrust (Newman et al., 2022). Rather, our findings seem to stem from the opposite disposition: to trust information.

Second, this inclination to trust information does not seem to stem from well-documented motivational and cognitive mechanisms, such as those pertaining to the compatibility (or lack thereof) of the evidence with one’s prior beliefs (Vicente et al., 2023; see also Edwards and Smith, 1996). It is, after all, quite unlikely that our participants had previously formed scientific hypotheses that could explain away their misgivings.

Third, previous studies show that people often engage in rationalizations for their own shortcomings, that is, they fabricate their own justifications (Shalvi et al., 2015). For instance, environmentally relevant behaviors have clear moral meaning (Berger, 2019; Griskevicius et al., 2010; Kennedy and Horne, 2019; Kohlova and Urban, 2020; Luomala et al., 2020; Puska, 2019; Puska et al., 2016; Urban et al., 2023). Consistent with this, people often make justifications for their inappropriate pro-environmental behaviors (e.g. rationalizing climate inaction; Wullenkord and Reese, 2021). Forming one’s own rationalization might stand in the way of effective self-deception, as a person might recognize that they are doing it for self-serving reasons (Sloman et al., 2010). However, a scientific explanation offered by others is likely to come across as less self-serving. The fact that other people are the ones doing the explaining grants impartiality to the explanation and is likely to boost its persuasiveness.

Moreover, scientific explanations might be particularly powerful because they come with the epistemic authority and credibility of expertise (Kruglanski et al., 2005; Petty et al., 1981; Pornpitakpan, 2004). Presumably, an explanation offered by scientific experts should have much more compelling exculpatory power than an explanation offered by laypeople (Kelman and Hovland, 1953). This need not involve the actual delivery of better explanations and arguments on the part of the experts; the mere appearance of scientificity is often sufficient to promote credibility (Tal and Wansink, 2016). So, while we believe that explanations other than scientific ones could have exculpatory power akin to that reported in this research, we also think that scientific explanations are particularly apt at alleviating guilt, namely because they are provided by (1) people other than the self and that (2) are ascribed epistemic authority (e.g. expertise).

To be sure, we do not wish to claim that science communication must always adopt a paternalistic approach, catering to people’s desires and motivations. There is evidence that people can also be made to change their mind in contentious domains (e.g. Wood and Porter, 2019). Key factors in such persuasion efforts include authority/expertise (e.g. Houck et al., 2024) and good arguments (Altay et al., 2022). However, research shows that even with good arguments (Dockendorff and Mercier, 2024) and the “best available evidence” (Rynes et al., 2018), people can remain unswayed. Thus, while the ultimate goal of science communication is not to massage egos and soothe consciences, when possible, science communication should avoid clashing with fundamental human motivations. Rather, as these studies suggest, science communication might even harness those basic drives to foster its efficacy.

One potential limitation of this research is that in Study 1 participants were psychology students. Indeed, psychology students might be more familiar with, or predisposed to accept, scientific explanations for people’s behavior. However, we would argue that including psychology students in this study only works against our hypothesis. Psychology students spend years learning that psychological science can explain a great deal of human behavior, which should predispose them (more than the general population) to believe that all sorts of behavior are explainable by science. However, the specific results pattern observed in Study 1 (with participants displaying greater belief in science in the condition where this is particularly self-serving) is not in line with this interpretation of training or selection biases. Rather, these results seem to align much better with a motivated reasoning account. And indeed, Study 2, whose participants are not psychology students but rather members of the general lay population, supports this account.

Summing up, these studies differ from previous research several ways. Our effects point to motivated acceptance of science, as opposed to the impressive amount of research already conducted on motivated rejection of science. This motivated reaction to scientific explanations does not seem to be guided by previous documented mechanisms, such as (in)compatibility with one’s previous beliefs, or a conspiratorial mistrustful mind-set. Rather, is most likely prompted by a desire to believe those explanations (as they are perceived as providing an excuse for one’s wrongdoings), and the persuasive power of explanations provided by other people who are experts (as opposed to rationalizations fabricated by the lay self). Finally, this research might suggest novel interventions to promote the acceptance of sound scientific information.

## Supplemental Material

sj-pdf-1-pus-10.1177_09636625241261320 – Supplemental material for Self-serving beliefs about science: Science justifies my weaknesses (but not other people’s) Supplemental material, sj-pdf-1-pus-10.1177_09636625241261320 for Self-serving beliefs about science: Science justifies my weaknesses (but not other people’s) by Francisco Cruz and André Mata in Public Understanding of Science
