# Supplementary material for: Self-serving beliefs about science: Science justifies my weaknesses (but not other people’s)
Source: Public Underst Sci. 2024 Jul 30;34(2):172–87. doi: 10.1177/09636625241261320 (PMC12989628; doi:10.1177/09636625241261320)
Supplement: sj-pdf-1-pus-10.1177_09636625241261320 – Supplemental material for Self-serving beliefs about science: Science justifies my weaknesses (but not other people’s) [file sj-pdf-1-pus-10.1177_09636625241261320.pdf]

Supplemental Materials

Self-Serving Beliefs about Science:

Science Justifies My Weaknesses (But Not Other People's)

by F. Cruz and A. Mata.

This supplement contains additional information on the statistical analyses conducted and on the materials used in the above-mentioned work.

TABLE OF CONTENTS

Table A1. Portuguese (i.e., original) wording of Study 1’s items.

Table A2. 2 (Valence) x 2 (Target) mixed ANOVA on explainability.

Table A3. 2 (Valence) x 2 (Target) mixed ANOVA on (im)materiality.

Table A4. Items used in Study 2a (adapted from Gudjonsson & Singh, 1989).

Table A5. Items used in Study 2b (and respective sources).

Table A1. Portuguese (i.e., original) wording of Study 1's items.

	Original (Portuguese)	Disclosed in the main text (English)
Explainability	*A ciência consegue explicar a ação que descrevi acima. Isto é, existe uma explicação científica para esta ação.*	*Science can explain the action I described above. That is, there is a scientific explanation for this action.*
	Os cientistas (e.g., neurocientistas, psicólogos, etc.) já têm a capacidade para compreender a razão do comportamento descrito acima.	*Scientists (e.g., neuroscientists, psychologists, etc.) are already capable of understanding the reason behind the behavior described above.*
	A ação que descrevi acima é facilmente explicada pela ciência.	*The action that I described above is easily explained by science.*
Materiality	*A ação que descreveu está associada a uma parte não material (e.g., mente, espírito, alma) ou material (e.g., cérebro) da sua pessoa?*	*Is the action you described associated with a non-material (e.g., mind, spirit, soul) or material (e.g., brain) part of [you/your acquaintance]?*

Table A2. 2 (Valence) x 2 (Target) mixed ANOVA on explainability.

Effect	*SS*	*df*	*MS*	*F*	*p*	η_p^2
Target (A)	4.10	1	4.10	0.64	.425	.01
Error	778.10	122	6.38			
Valence (B)	13.80	1	13.80	10.99	.001	.08
Error	153.28	122	1.26			
A x B	5.58	1	5.58	4.42	.037	.04

Table A3. 2 (Valence) x 2 (Target) mixed ANOVA on (im)materiality.

Effect	*SS*	*df*	*MS*	*F*	*p*	η_p^2

Target (A)	10.07	1	10.07	1.83	.179	.02
Error	672.04	122	5.51			
Valence (B)	23.87	1	23.87	11.44	< .001	.09
Error	254.59	122	2.09			
A x B	0.00	1	0.00	0.00	.982	.00

Table A4. Items used in Study 2a (adapted from Gudjonsson & Singh, 1989)

Subscale	Item
Guilt	*“1 – You should feel much* **less** *ashamed of what you did” to*<br>*“9 – You should feel much* **more** *ashamed of what you did”*<br><br>*“1 – You should feel much* **less** *remorse/guilt for what you did” to*<br>*“9 – You should feel much* **more** *remorse/guilt for what you did”*<br><br>*“1 – You should punish yourself a lot* **less** *for what you did” to*<br>*“9 – You should punish yourself a lot* **more** *for what you did”*<br><br>*“1 – You deserve to be much* **less** *severely punished for what you did” to*<br>*“9 – You deserve to be much* **more** *severely punished for what you did”*
External Attribution	*“1 – You should blame yourself much* **less** *” to*<br>*“9 – You should blame yourself much* **more** *”*<br><br>*“1 – You should receive a much* **smaller** *punishment for what you did” to*<br>*“9 – You should receive a much* **greater** *punishment for what you did”*<br><br>*“1 – You* **did not have** *good reasons for acting the way you did” to*<br>*“9 – You* **had very** *good reasons for acting the way you did” **<br><br>*“1 – You have much* **less** *excuse for what you did” to*<br>*“9 – You have much* **more** *excuse for what you did” **
Control	*“1 – It was totally* **under** *your control” to*<br>*“9 – It was totally* **beyond** *your control” **<br><br>*“1 – You were* **not at all in control** *of your actions” to*<br>*“9 – You were* **in full control** *of your actions”*<br><br>*“1 – You could* **not have avoided** *what you did” to*<br>*“9 – You could* **have easily had avoided** *what you did”*

Note: *Reverse-coded.

Table A5. Items used in Study 2b (and respective sources)

Subscale	Adapted from	Item
Guilt	Gudjonsson and Singh (1989)	*"1 - You should feel much* **less** *ashamed of what you did"* to "*9 - You should feel much* **more** *ashamed of what you did"*
		"1 - You should feel much **less** *remorse/guilt for what you did"* to "*9 - You should feel much* **more** *remorse/guilt for what you did"*
		"1 - You should punish yourself a lot **less** *for what you did"* to "*9 - You should punish yourself a lot* **more** *for what you did"*
		"1 - You deserve to be much **less** *severely punished for what you did"* to *"9 - You deserve to be much* **more** *severely punished for what you did"*
		"1 - You should ***totally*** *forgive yourself for what you did"* to *"9 - You should* ***never*** *forgive yourself for what you did"*
		"1 - You have **much less** *reason to hate yourself for what you did"* to *"9 - You have* **all the more** *reason to hate yourself for what you did"*
External Attribution	Russell (1982)	*"1 - That what you did* ***reflects*** *an aspect of yourself"* to *"9 - That what you did* ***does not reflect*** *an aspect of yourself*" *****
		"1 - That what you did was caused by something ***outside*** *of you"* to *"9 - That what you did was caused by something* ***inside*** *of you"*
		"1 - That what you did **was** *caused by something about you"* to *"9 - That what you did* **was not** *caused by something about you"* *****
	Tamborini et al. (2018)	*"1 - That your behavior in this situation was caused by* ***your personal qualities****"* to "*9 - That your behavior in this situation was caused by* ***something beyond your personal qualities***" *****
		"1 - That what you did ***reflects*** *the real you"* to *"9 - That what you did* ***does not reflect*** *the real you"* *****
	Cucchi and Cavazza (2021)	*"1 - That the responsibility for what happened is to be attributed* ***to the particular circumstances in which you found yourself at that moment****"* to *"9 - That the responsibility for what happened is to be attributed* ***to yourself****"*
Control	McAuley et al. (1992)	*"1 - That what you did* **was** *manageable by you"* to *"9 - That what you did* **was not** *manageable by you"* *****
		"1 - You **could not** *regulate what you did"* to *"9 - You* **could** *regulate what you did"*

	*“1 - That what happened was something over which you **had no** power”* to<br>*“9 - That what happened was something over which you **had** power”*
Russell (1982)	*“1 - That what you did was **uncontrollable”*** **to**<br>*“9 - That what you did was **controllable**”*<br><br>*“1 - That what you did was **intended**”* to<br>*“9 - That what you did was **unintended**”* *****<br><br>*“1 - That you **are** responsible for what you did”* to<br>*“9 - That you **are not** responsible for what you did*” *****
Nadelhoffer et al. (2014)	*“1 - That you **did not have** the ability to do otherwise in this situation”* to<br>*“9 - That you **had** the ability to do otherwise in this situation”*<br><br>*“1 - That you **did not have** free will in this situation”* to<br>*“9 - That you **had** free will in this situation” **<br><br>*“1 - That the way the situation unfolded **was completely** up to you”* to<br>*“9 - That the way the situation unfolded **was not** up to you”* *****<br><br>*“1 - That ultimately you **had complete** control over your decisions and actions in this situation”* to<br>*“9 - That ultimately you **had no** control over your decisions and actions in this situation”* *****

Note: *Reverse-coded.
